# Microbial Safety of Smoothie Drinks from Fresh Bars Collected in Slovakia

**DOI:** 10.3390/foods10030551

**Published:** 2021-03-07

**Authors:** Monika Krahulcová, Barbora Micajová, Petra Olejníková, Klára Cverenkárová, Lucia Bírošová

**Affiliations:** 1 Faculty of Chemical and Food Technology, Department of Nutrition and Food Quality Assessment, Slovak University of Technology, Radlinského 9, 81237 Bratislava, Slovakia; baskam@ymail.com (B.M.); klara.cverenkarova@stuba.sk (K.C.); lucia.birosova@stuba.sk (L.B.); 2 Faculty of Chemical and Food Technology, Institute of Biochemistry and Microbiology, Slovak University of Technology, Radlinského 9, 81237 Bratislava, Slovakia; petra.olejnikova@stuba.sk

**Keywords:** coliform bacteria, enterococci, smoothie, antibiotic resistance

## Abstract

Among the many consumers in Slovakia, smoothies are nowadays gaining popularity. Smoothie drinks are prepared from raw fruits and vegetables. Therefore, their microbiological safety depends on hygiene standards. The aim of this work was to monitor and quantify selected sensitive and antibiotic-resistant microorganisms present in collected smoothies. Twenty analyzed smoothie samples were collected from six food service establishments (fresh bars) in the capital city of Slovakia, Bratislava. Antibiotic-resistant bacteria were found in at least one of each fresh bar. Antibiotic-resistant coliform bacteria prevailed, especially in green smoothies or juices containing more vegetable ingredients. Resistance to ampicillin, ciprofloxacin, tetracycline, chloramphenicol, and gentamicin was observed in the case of coliform bacteria. More than half of the smoothie drink samples did not contain resistant enterococci. On the other hand, vancomycin-resistant enterococci were detected in 20% of samples. The most frequently isolated antibiotic-resistant strains belonged to the *Enterobacter* spp. or *Klebsiella* spp. genus. In the last part of the work, the pretreatment effect of smoothie components on the selected microorganisms’ counts in the final product was investigated. Washing ingredients with an aqueous solution of a biocide agent containing silver and hydrogen peroxide proved to be the most effective way to decrease bacterial counts.

## 1. Introduction

Fresh fruits and vegetables represent essential components of a healthy human diet. The protective role of these ingredients against the most prevalent diseases of civilization, such as diabetes, obesity, hypertension, or coronary heart disease, has been described in many studies [1,2,3,4,5]. Their beneficial effect is based on the rich composition of many vitamins, minerals, dietetic fiber, and phytochemicals. For example, carotenoids may prevent oxidative stress caused by reactive oxygen species, which damage cell membranes or DNA [4,6]. Dietetic fiber has a significant impact on the gut microbiome [7,8], and its consumption may reduce the risk of colon cancer development [9,10]. Many people prefer a healthier lifestyle with regard to the consumption of fresh and healthy food. As a result, the demand for fresh fruits and vegetables, as well as smoothies, is increasing. This has caused the expansion of minimally processed ready-to-eat or ready-to-use food in retail [11]. Despite their demonstrated positive effect on various chronic human diseases, fresh fruits and vegetables have recently become the cause of foodborne illness outbreaks in developed countries [12].

According to a study conducted by Callejo et al. (2015), who summarized reported foodborne outbreaks in the United States (US) and the European Union (EU) between 2004 and 2012, *Salmonella* was the most prevalent bacterial pathogen [13]. The second most common bacterial pathogen was *E. coli* [13]. One of the biggest outbreaks was linked to the consumption of spinach in the US in 2006. Almost 200 people were infected by the *E. coli* O157:H7 strain, and three deaths were confirmed [14]. In 2016, a large outbreak of *E. coli* O157 appeared in the United Kingdom, where the outbreak vehicle was mixed salad leaves [15]. In 2020, two multistate outbreaks were detected in the US. The first was linked to leafy greens contaminated by *E. coli* O157:H7 [16], and the second, more serious outbreak was caused by the same strain found in Romaine lettuce [17]. As a result, fifteen people developed hemolytic uremic syndrome, a type of kidney failure [17].

Smoothie drinks are prepared from a wide range of raw fruits and vegetables, using a blender. According to previous findings, they also present a potential threat to the occurrence of various pathogenic microorganisms. One of the reported outbreaks was smoothie drinks contaminated with hepatitis A in 2016. The Centers for Disease Control and Prevention (CDC) recorded 134 people with hepatitis A after drinking a smoothie containing strawberries imported from Egypt [18]. Although the *Enterobacteriaceae* family includes mostly harmless bacteria, some of them contribute to food spoilage or exist as foodborne pathogens [19]. Coliform bacteria belong to the *Enterobacteriaceae* family and are commonly found as a natural part of the intestine microbiome (*E. coli*). On the other hand, coliforms can be presented in soil or water (*Serratia* spp., *Enterobacter* spp., and *Klebsiella* spp.) [20]. Coliform bacteria and enterococci have mostly been used as indicators of postprocessing contamination in foodstuffs, although not all of these organisms have origins in the intestine of humans and animals [21,22]. However, their occurrence may represent significant mishandling during the manipulation of smoothie ingredients. It could be related to bad hygiene practice due to contaminated equipment use during preparation (hygiene of food contact surfaces) or the personal behavior of the worker such as lack of handwashing [23,24].

Besides the possible presence of foodborne pathogens in ready-to-eat plant food, another growing food safety concern is related to the role of food in human exposure to antibiotic-resistant bacteria as a reservoir of resistance genes [11]. The risk of the bacterial contamination of plant origin food can occur through fertilizers with animal manure, soil, and irrigation water, as well as by washing, handling, and processing vegetables or fruit during the postharvest period [25,26]. Commensal *Enterobacteriaceae* in fresh fruit and vegetables could pose a serious issue regarding the spread of antimicrobial resistance among sensitive and resistant strains [27,28]. Some of them previously caused outbreaks of foodborne diseases [19,29]; for example, the contamination of sprouts by Shiga-toxin-producing *E. coli* (STEC) producing extended-spectrum β-lactamase (ESBL) caused the outbreak in Germany in 2011 [30]. Consequently, ESBL-producing *Enterobacteriaceae* in vegetables have been reported in several countries, such as multidrug-resistant *Klebsiella pneumoniae*, with variants of CTX-M enzymes in Southeast Asia [31]; ESBL-producing *E. coli*, *Enterobacter* spp., or *K. pneumoniae* reported in China [32] and Switzerland [33]; or ESBL-producing *K. pneumoniae* reported in North Africa [34].

The number of retail locations selling fresh smoothie drinks in Slovakia is increasing. The consumption of these drinks is increasing as well. On the other hand, smoothie drinks are ready-to-eat products, and their microbial safety is very important for public health. The dissemination of antimicrobial resistance in food chains in Slovakia is monitored mainly in foods of animal origin. Therefore, more data about antibiotic-resistant bacteria occurrence in plant food are needed.

In the present study, selected microorganisms (coliform bacteria, enterococci, yeasts, and fungi) in smoothies of fruits and vegetable origin from different food service establishments (FSEs) in Bratislava (Slovakia) were quantitatively examined for the first time. In the case of coliform bacteria and enterococci, the occurrence of antibiotic-resistant bacteria was also assessed. Resistant coliform bacteria and resistant enterococci were subsequently isolated and identified. The effects of pretreatment by washing the used smoothie ingredients on microbial elimination in smoothie drinks were also assessed.

## 2. Materials and Methods

### 2.1. Sample Collection

Samples of twenty different smoothies were collected from six FSEs in Bratislava (Slovakia). The types of smoothies were chosen according to their composition and consumer preferences. Smoothie samples were labeled with an abbreviation, as summarized in Table 1. The first letter of the abbreviation marks the main component of the smoothie, and the second letter indicates which fresh bar served that smoothie. Smoothies were named by their main ingredient, such as fruit (F) or green (G). For example, GA1 means a green smoothie served in Fresh Bar A. Number one marks that it is the first obtained sample of green smoothies (GA2—second green smoothie from Fresh Bar A). Seven samples of green smoothies and thirteen samples of fruit smoothies were processed. The predominance of fruit drinks was based on the statement of the seller as the effort was to analyze the best-selling drinks (Table 1).

### 2.2. Microbiological Analysis

Samples from fresh bars in sealed bottles or cups were immediately transported to the laboratory for microbial analysis. Samples were not cooled during collection, and all samples were transported to the laboratory within 50 min of collection. Series of dilutions from 10^−1^ to 10^−5^ were prepared in phosphate buffer saline. Microbiological analysis focused on aerobes, yeasts, and fungi to explain the general microbial load of the samples. European legislation states a detection limit for smoothie foods only for the occurrence of *E. coli*. According to Commission Regulation (EC) No. 2073/2005 of 15 November 2005 on microbiological criteria for foodstuffs for unpasteurized fruits and vegetable juices (ready-to-eat), *E. coli* microorganisms cannot exceed 1000 CFU per 1 mL of the sample [35]. Coliform bacteria and enterococci detected in the smoothie samples were chosen as microbial indicators of proper hygiene according to the widely used procedure of fertilization with manure as a source of contamination of raw products. Appropriate dilutions were spread on nonselective and selective agar plates:

- Plate count (PC) agar (BioLife Italiana srl., Milan, Italy) for aerobes;
- Yeast extract glucose chloramphenicol (YGC) agar (OXOID Deutschland GmBH., Wesel, Germany) for yeasts and fungi;
- Chromocult coliform (CC) agar (VWR, Darmstadt, Germany) for coliform bacteria;
- Slanetz–Bratley (SB) agar (BioLife Italiana srl., Milan, Italy) for enterococci.

Agar plates with PC and CC were cultivated aerobically at 37 °C for 24 h. SB plates were cultivated aerobically at 40 °C for 48 h and YGC at 25 °C for 5 days. The results were expressed as the mean of all the repetitions together with the standard deviation (SD).

### 2.3. Monitoring of Antibiotic-Resistant Bacteria

For the monitoring and selection of antibiotic-resistant coliform bacteria, different concentrations of antibiotics, such as ampicillin, gentamicin, chloramphenicol, ciprofloxacin, and tetracycline (Sigma-Aldrich, St. Louis, MO, USA), were added into cooled CC media. Agar was mixed with each antibiotic and poured into plates under sterile conditions. The monitoring of enterococci was performed similarly using these antibiotics: ampicillin, gentamicin, ciprofloxacin, and vancomycin (Sigma-Aldrich, St. Louis, MO, USA). Antibiotics were mixed with cooled SB media. Individual antibiotics were selected to cover the largest possible range of drug classes (beta-lactams, aminoglycosides, miscellaneous agents, fluoroquinolones, glycopeptides, and tetracyclines). Plates of CC and SB agar with the addition of antibiotics were determined to screen resistant coliform bacteria and enterococci in the smoothie samples. The concentrations of the applied antibiotics are shown in Table 2. Each antibiotic concentration was chosen according to its resistance limits established by the European Committee on Antimicrobial Susceptibility Testing (EUCAST) [36], except for tetracycline, which is not established by European guidelines for coliforms. The concentration of tetracycline was applied according to American resistance limit given by Clinical and Laboratory Standards Institute (CLSI) guideline [37]. *E. coli* collection culture (CCM 3988) was used as a control. The results were expressed as the mean of all the repetitions together with the standard deviation (SD).

### 2.4. Identification of Isolated Antibiotic-Resistant Coliform Bacteria and Enterococci

Antibiotic-resistant bacteria were isolated from CC agar (VWR, Darmstadt, Germany), supplemented with antibiotics, and inoculated on Mueller–Hinton agar (BioLife Italiana srl., Milan, Italy) plates. The quadrant streaking plate technique was used to achieve a pure culture. A presterilized loop spread the isolate across one-quarter of the agar surface. This action was repeated for each of the four quadrants of the plate, and the metal loop was sterilized using a flame prior to contact with the agar medium [38]. After incubation of the isolates on Mueller–Hinton agar (24 h, 37 °C), each grown colony was identified by matrix-assisted laser desorption/ionization–time of flight (MALDI–TOF) mass spectrometry analysis. A sample for analysis was prepared by mixing a fresh isolated culture of bacteria spotted onto a steel target plate (Bruker Daltonics Inc. Billerica, MA, USA) and an energy absorbent, known as the matrix (α-cyano-4-hydroxycinnamic; Bruker Daltonics Inc. Billerica, MA, USA). The matrix was used in a volume of 1 μL (a saturated solution in 50% acetonitrile and 2.5% trifluoroacetic acid). The mixture of fresh isolate and matrix was dried at room temperature. The sample is entrapped within the energy absorbent (matrix) in the mixture and cocrystallizes during the drying process. These unknown bacteria were identified with an AutoFlex I TOF-TOF apparatus (Bruker Daltonics Inc. Billerica, MA, USA) in a linear positive-ion mode across the m/z range of 2.000 to 20.000, with the gating of ions below m/z 400 and a delayed extraction time of 450 ns. Each spot was measured using 2.000 laser shots at 25 Hz in groups of 50 shots per sampling area of the spot. The data sampling rate was 0.5 GHz. Each plate was calibrated using a Bacterial Test Standard (Bruker Daltonics). Based on the TOF information, a characteristic spectrum was generated using MALDI BioTyper software (version 2.0) (BioTyper Library version 3.0; Bruker Daltonics). The algorithm for spectral pattern matching resulted in a logarithmic score from 0 to 3 (a score of >1.9—species identification, a score of 1.7 to 1.9—genus identification, a score of <1.7—no identification).

### 2.5. Efficiency of Prewashing on Bacterial Count Reduction

The green smoothie consisted of orange (60 g), apple (30 g), spinach (10 g), and drinking water (250 mL). It was prepared under laboratory conditions to simulate “homemade” smoothies. The smoothie was prepared in three different ways (control sample, first experiment, and second experiment), and the bacterial count was performed in triplicate and repeated three times. The ingredients used in the control sample were not washed. The ingredients in the first experiment were prewashed thoroughly under drinking tap water only as it is the main step in the cleaning process of ingredients at home. In the second experiment, Sanosil Super 25 Ag (Sanosil Ltd., Hombrechtikon, Switzerland) disinfectant, consisting of 50% hydrogen peroxide and 0.05% silver, was added to the washing solution. Hydrogen peroxide is approved as an antimicrobial agent for use in the food industry in Europe [39]. It has a direct toxic effect on many bacterial species, and Sanosil biocide has shown to be highly effective against pathogenic bacteria, as well as fungi, amoeba, and viruses (such as Norovirus) [40]. The use of this disinfectant in the food industry is compliant with Hazard Analysis and Critical Control Point (HACCP) guidelines, which ensures safe and proper cleaning and disinfection in the food industry. The HACCP plan in the food hygiene control system focuses on identifying the specific health risk of individual foods, which sets out a preventive measure to control the potential risk [41]. The concentration was applied according to the manufacturer’s recommendation: 500 mL of drinking water was added to 200 μL of disinfectant. The smoothie ingredients were treated with this solution for 60 min (according to the manufacturer’s recommendation) and then rinsed under running tap water. The smoothie was subsequently prepared in a smoothie mixer (SENCOR SBL 2210WH Japan) with an energy consumption of 500 W. The smoothie bottles were sterilized in the form of saturated steam under pressure before each experiment (121 °C, 23 min, 120 kPa). The mixing time was 1 min for all variants. In each sample, coliform bacteria, enterococci, fungi, and yeasts were detected, according to Section 2.1 and Section 2.2, as the samples collected in fresh bars. Data were treated with a one-way ANOVA test and a least significant difference of 95%.

## 3. Results and Discussion

### 3.1. Occurrence of Selected Microorganisms in Smoothies

Fruits and vegetables used for smoothie preparation have been repeatedly described as potential sources of many different microorganisms and, together with secondary microbial contamination, may pose a risk for consumers [42,43,44,45]. In the first part of this work, the monitoring of the prevalence of total fungi, yeasts, and aerobic bacteria (considering coliforms and enterococci) was performed. Table 3 shows the quantitative representation of the mentioned microorganisms in the studied smoothie samples. Total aerobic bacteria, yeasts, and fungi were monitored for a general view of microorganism load in the smoothie samples. The number of total aerobic bacteria ranged from 2.9 to 7.3 log CFU/mL. The results correlate with the bacterial population level in fruits and vegetables, which ranged from 3 to 7 log CFU/g, according to the type of ingredient [46,47,48]. The occurrence of yeasts and fungi was 1.8 log CFU/mL to higher than 3.0 log CFU/mL. One sample of green smoothie prepared from spinach, pear, and banana (GF1) did not contain any yeasts and fungi (Table 3). The prevalence of yeasts and fungi in the smoothie samples was similar to other studies, where the numbers oscillated around 2.8 [49] and 2.17 log CFU/mL [50]. The highest occurrence of yeasts and fungi was detected in all samples obtained from Fresh Bar D (GD1—spinach, orange, carrot, banana; FD1—kiwi, orange, banana; FD2—strawberries, banana, apple) and Fresh Bar E (FE1—apple, cucumber, spinach; FE2—pear, beetroot, carrot, celery; FE3—grapefruit, orange, ginger). This could be due to the predominance of fruit smoothies. Fruits are more likely to be contaminated by molds and yeasts due to their composition, which is rich in sugar and other nutrients [50,51]. The origin of contamination could be via air, as both of the fresh bars were located inside shopping centers as open-space shops. It needs to be noted that air can play an important role in the transfer of spoilage microorganisms [52,53]. The second collected fruit smoothie from Fresh Bar F has also a higher occurrence of yeasts and fungi (FF1—strawberries, banana, orange) (Table 3).

Coliform bacteria and enterococci could be considered indicator bacteria, pointing out the hygienic process of the smoothie preparation. The consumption of raw and thermally untreated foods contaminated with such bacteria may cause the spread of disease in humans [54]. Coliform bacteria were detected in all tested smoothie drinks. Korir et al. (2016) registered total coliform bacteria in 38.7% of different fresh vegetables (414 samples) [47]. The number of coliforms in the green smoothie samples ranged from 2.1 to 3.7 log CFU/mL. This difference could be caused by the limited number of samples as well as better manufacturing praxis and hygiene.

In one smoothie sample, the occurrence of *E. coli* was observed. It was the green smoothie marked as GA1, which was composed of spinach, banana, melon, and lime. Previous research revealed that more diseases are connected to the consumption of contaminated leafy vegetables; therefore, green smoothies could carry a higher risk of containing potential bacterial pathogens such as *E. coli*, as it was in this case [55]. The sample marked as GA1 contained 150 CFU/mL of *E. coli*. Consequently, the number of microorganisms was still at a satisfactory level following Commission Regulation (EC) No. 2073/2005, which was mentioned above (1000 CFU/mL) [35].

Total enterococci were observed in all fresh bars examined. They were not detected in two samples of fruit smoothies. The first was collected from Fresh Bar C and was composed of banana, strawberries, and apple (FC2). The second was collected from Fresh Bar D, and the sample obtained kiwi, orange, and banana (FD1). The remaining eighteen smoothie samples (90%) were positive for the presence of enterococci. The results are slightly related to the study performed by Sun et al. (2021), where total enterococci were detected in 87.1% of vegetables, 100% of our green smoothies, and 59.8% of fruits (84.6% of our fruit smoothies) [31]. Enterococci, a fecal-contaminated bacteria, can be mainly transferred to fruits and vegetables through irrigated water, organic fertilization of animal origin [25,56], or, additionally, in the case of smoothies, through low hygiene standards of personnel working with food or contact with surfaces [57]. The difference between the green and fruit smoothies in the occurrence of total enterococci was insignificant as there was a disproportion of each type of smoothie, and the offer and demand of the fruit smoothies were greater than the green smoothies. The number of *Enterococcus* spp. ranged from 1.1 to 2.9 log CFU/mL.

Many studies showed that the microbial contamination of ready-to-eat foods can cause health problems among consumers [58,59,60,61]. An example of this untreated food is the smoothie. It could be prepared at the time of selling with asymptomatic food handlers as potential carriers of microbes or with handlers with poor personal hygienic standards [45,62].

### 3.2. Occurrence of Antibiotic-Resistant Coliform Bacteria and Enterococci

A growing food safety concern is related to the role of food in human exposure to antimicrobial-resistant bacteria, including zoonotic, commensal, and environmental bacteria as resistance genes reservoirs [63,64,65]. Commensal *Enterobacteriaceae* in fresh fruit and vegetables may represent a source of transmissible antimicrobial resistance genes [27,28,66]. According to this, one of the aims of this work was to study the occurrence of antibiotic-resistant coliform bacteria and enterococci in the studied smoothie samples (Table 4 and Table 5). Antibiotics were chosen as representatives of different antibiotic classes. The concentrations were applied according to the resistance thresholds given by EUCAST and CLSI [36,37]. Table 4 presents antibiotic-resistant coliforms, which were not detected in all fruit smoothies collected from Fresh Bar A (FA1—melon, apple, pear, mint; FA2—pineapple, orange, coconut milk) and in one green smoothie obtained from Fresh Bar B (GB1—avocado, mango, spinach, apple) (Table 4).

Ampicillin-resistant coliform bacteria have been registered in most smoothies. This could be due to the intrinsic resistance of most coliform bacteria to this antibiotic [67]. In green smoothie samples from FSE A (GA1—spinach, banana, melon, lime) and fruit smoothie from FSE E (FE2—pear, beetroot, carrot, celery), the occurrence of both tetracycline- and chloramphenicol-resistant coliforms were observed. Tetracycline resistance was registered only in three smoothies (15%) as it was also detected in green smoothies from FSE B (GB2—apple, orange, carrot, celery, ginger). Resistance to chloramphenicol was also detected in fruit smoothies from FSE B (FB2—orange, apple, blueberries, strawberries) and D (FD1—kiwi, orange, banana) and in all samples taken from FSE F (GF1—spinach, pear, banana; FF1—strawberries, banana, orange). Altogether, chloramphenicol-resistant coliform bacteria were detected in 30% of all smoothie samples. Although the use of chloramphenicol in clinical praxis in Slovakia has been delimited [67], the appearance of resistance to this antibiotic could be the consequence of cross-resistance. Gentamicin resistance was detected in 45% of collected smoothie samples, specifically in one sample from Fresh Bar C (FC3—blueberries, raspberries, strawberries, pineapple, apple) and in all collected samples from FSE D–F. The worst situation in the case of the occurrence of antibiotic-resistant coliform bacteria was observed in smoothies from Fresh Bar F (GF1—spinach, pear, banana; FF1—strawberries, banana, orange), where was not observed only resistance to tetracycline. In both green and fruit smoothies, high numbers of ampicillin, ciprofloxacin-, gentamicin-, and chloramphenicol-resistant coliforms were detected.

Resistant enterococci occurred in nine types (45%) of smoothies from all twenty smoothie samples from the FSEs (Table 5).

Antibiotic-resistant enterococci were not detected in 55% of all smoothie samples. Fresh Bar C (GC1—spinach, mango, banana, orange; FC1—strawberries, mint, lime, apple; FC2—banana, strawberries, apple, FC3—blueberries, raspberries, strawberries, pineapple, apple) did not contain smoothies with the presence of antibiotic-resistant enterococci. Both fruit smoothies collected from Fresh Bar B (FB1—strawberries, acai, chia seeds, orange, apple; FB2—orange, apple, blueberries, strawberries) also did not contain antibiotic-resistant enterococci. Similarly, to coliform bacteria, a high abundance of ampicillin-resistant enterococci was determined. Ciprofloxacin-resistant enterococci were observed only in one fruit smoothie sample from Fresh Bar F (FF1—strawberries, banana, orange).

In four samples (20%) collected from three of the six tested fresh bars (GA1—spinach, banana, melon, lime; FA1—melon, apple, pear, mint; GB2—apple, orange, carrot, celery, ginger; FE3—grapefruit, orange, ginger), vancomycin-resistant enterococci (VRE) were registered. These bacteria were first identified in the mid-1980s and spread rapidly. They are currently a major problem in many healthcare institutions worldwide [68]. In 2017, the WHO published a list of bacteria for which new antibiotics are urgently needed. VRE belong to the second group of resistant bacteria with a high priority [69]. Torre et al. (2010) found VRE in one-third of analyzed fresh fruit and vegetable, especially in curly endive and lettuce [70]. According to the irregular presence of VRE in these fresh bars, it could be assumed that their occurrence is affected by one specific smoothie ingredient. For example, in the case of the GB2 smoothie sample, it could be due to using celery, as was presented in another study [70]. On the other hand, there is also a possibility of transfer due to inappropriate hygiene and handling during the drink preparation [71].

Antibiotic resistance among coliform bacteria was more prevalent compared to antibiotic resistance among enterococci. It could be due to the higher initial microbial load of coliforms in the studied smoothie samples.

### 3.3. Identification of Antibiotic-Resistant Coliform Bacteria

**Figure 1:**
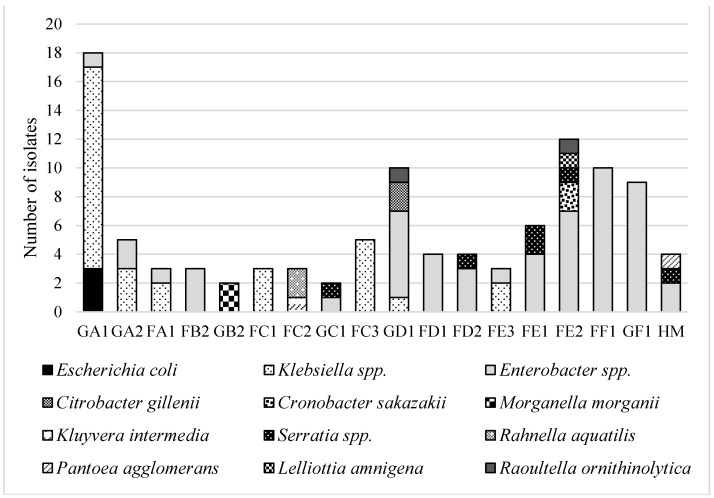
Identified antibiotic-resistant coliform bacteria in smoothies collected from fresh bars in Bratislava.

Individual resistant strains were chosen from all used agar plates supplemented randomly with antibiotics. From all smoothie samples, 102 isolates were successfully isolated and identified (Figure 1). Isolates were transferred from diagnostic media with an antibiotic to Mueller–Hinton media and consequently identified by a MALDI–TOF biotyper.

Most strains belonged to the *Enterobacter* genus (52 strains of all isolated bacteria), predominantly isolated from plates with the addition of gentamicin (50%) and chloramphenicol (25%). In the case of *Enterobacter* spp., most strains were found in fruit smoothies (61%), and the rest of this gender was detected in green smoothies (39%). The *Enterobacter* spp. genus is predominantly habituated in soil and water and is often isolated from fruit and vegetable samples [72]. However, this microorganism may also be found in human feces or the respiratory tract of men. *Enterobacter* spp. has been noticed as a cause of urinary tract infection in hospital environments. This identification showed strains of *E. cloacae*, *E. asburiae*, and *E. ludwigii*. *Enterobacter* spp. are also associated with bacteremia but more in comparison to *Klebsiella* spp. [73]. *Klebsiella* species (30 stains of all isolated bacteria) were found in green as well as in fruit smoothies. The majority of *Klebsiella* spp. was identified as *K. pneumoniae* (60%) and predominantly was isolated from gentamicin plates (50%) and ampicillin plates (44%). This bacterium could represent a higher risk for staff preparing smoothies. It can be inhaled in the form of a bioaerosol produced during preparation and cause bronchopneumonia or bronchitis [74]. *K. oxytoca* formed the rest of the isolated bacteria and was isolated from gentamicin (58%) and ampicillin (42%). Other coliforms were identified, such as *E. coli*, *Serratia liquefaciens* and *Serratia odorifera*, *Morganella morganii*, *Citrobacter gillenii*, *Cronobacter sakazakii*, *Raoultella ornithinolytica*, *Kluyvera intermedia*, and *Lelliottia amnigena*. All isolates identified as *E. coli* were ampicillin resistant. Two isolates were identified as *Rahnella aquatilis*, which is rarely reported as a human pathogen. *Rahnella* spp. is mainly the cause of infections between immunocompromised patients [75]. The mentioned bacterium has been found in freshwater, soil, and certain animals, such as snails and certain beetles [76], so the possible source in smoothies can be leafy vegetables or fruits that do not require peeling (in our case strawberries).

### 3.4. Efficiency of Washing the Raw Materials Used in Smoothies

Smoothie drinks and other ready-to-eat products are particularly important because of the microbial load of ingredients used during preparation as they are consumed without any heat treatment and therefore the present microorganisms are not devitalized. Proper treatment of the raw material is an important step in smoothie preparation. Ingredients used to prepare most fruit smoothies are mainly cleaned by removing the surface skin/peel, which could also eliminate a significant proportion of microorganisms. On the other hand, the decontamination of vegetables is much more complicated because vegetables are contaminated mainly by soil deposits, containing a wide range of bacteria. The external tissue inhabits a high number of different bacteria in fresh produce plants. In addition to this fact, bacteria have been detected within plant tissue [77,78]. It is very complicated to eliminate such endophytic bacteria by washing as it stays inside the plant [79]. According to this, another aim of this work was to show how the proper washing of smoothie components can contribute to a decrease in the final microbial count in smoothie drinks (Table 6).

As a control, a smoothie from unwashed components was prepared. The second sample consisted of smoothie components washed under a tap with running drinking water. The ingredients in the third smoothie sample (WS) were treated with an aqueous solution of commercial Sanosil disinfectant. From the one-way ANOVA analysis, we could conclude that treatments had a different effect on the monitored microorganisms. The highest number of all detected microorganisms was detected in the control sample. Washing the raw materials under running drinking water led to a reduction of the microbial count by less than one logarithmic order. The results relate to Falomir et al. (2010), who found out that washing ingredients with drinking water had an insignificant effect on the population of coliform bacteria [80]. They surmise different mechanisms of adherence for these bacteria on plant surfaces, which could be responsible for this insignificant effect of cleaning ingredients under running drinking water [80]. Washing raw materials also did not lead to the detection of the number of coliform bacteria resistant to ciprofloxacin. On the other hand, treatment with Sanosil solution had the best effect in decreasing the bacterial count. The WS sample contained the lowest number of total aerobic and coliform bacteria compared to the control sample and water-treated sample. The effect on coliform bacteria could be due to silver ions obtained in biocides, as Gram-negative species appear to be more sensitive compared to Gram-positive bacteria. According to research conducted by Silvestry-Rodriguez et al. (2007), silver ions have the potential to bind to negatively charged peptidoglycans located at the cell wall [81].

The biocide agent Sanosil had a weak effect on the number of yeasts and fungi compared to the control sample. In the case of ampicillin-resistant bacteria, they were constantly decreased by the addition of washing. The WS sample decreased most of the monitored microorganisms, except ciprofloxacin-resistant coliform bacteria. Our results correlated with other studies with the use of detergent to eliminate bacteria [82,83]. The Sanosil biocide agent was the most effective, but as other studies have shown, efficiency is most likely related to microbial gender or the number of bacterial quantities, concentration of hydrogen peroxide in the solution, time of exposure, and many other factors [39,82,83,84].

Although this experiment was mainly demonstrative and focused on washing and sanitation using biocide Sanosil, it is important that other factors also affect the microbial safety of smoothie drinks. To establish a definitive correlation between cleaning methods and microbial quality, more variants with different microorganisms, different vegetables, fruits, and disinfection agents would need to be analyzed.

## 4. Conclusions

Currently, the demand of consumers for healthy food is gaining strength. Smoothies prepared from fresh fruit and vegetables and contain nutrients that could support the rapid growth of foodborne bacterial pathogens. This paper proves that smoothie drinks sold in FSEs contain micromycetes, yeasts, and bacteria, including coliforms and enterococci, which, after additional investigation, include bacteria such as *Enterobacter* spp., *Klebsiella* spp., or *E. coli*. Moreover, antibiotic-resistant bacteria were present in all tested fresh bars and contribute to antibiotic resistance dissemination in the food chain. Antibiotic-resistant bacteria may create a risk, especially for immunocompromised people. The presence of different microorganisms has a high impact on human health; for instance, through bioaerosols generated during preparation and handling. It can also endanger personnel through the inhalation of dangerous bacteria such as *Klebsiella pneumoniae*, which was present in the collected smoothie samples. Reducing the risk of exposure to enteric pathogens can be through strict adherence to recommended good hygienic practice and hygiene levels for smoothie preparation. It will also reduce the risk of the transfer of antibiotic-resistant bacteria. One of the conclusions was that washing smoothie ingredients with a biocide agent may have an effect on microbial composition, although data depend on many incoming variables.

There is no published study on the microbial safety of smoothie drinks in Slovakia. Moreover, the number of studies focusing on the microbial loads of smoothie samples is very low because the results obtained in this study could not be compared with such data. Our results point out that the occurrence of antibiotic resistance in the food chain can be a serious issue.

## Figures and Tables

**Table 1:** Name and composition of the analyzed smoothie samples.

**Fresh Bar A**
GA1: Spinach, banana, melon, lime
GA2: Broccoli, ice lettuce, cucumber, green barley, apple juice
FA1: Melon, apple, pear, mint
FA2: Pineapple, orange, coconut milk
**Fresh Bar B**
GB1: Avocado, mango, spinach, apple
GB2: Apple, orange, carrot, celery, ginger
FB1: Strawberries, acai, chia seeds, orange, apple
FB2: Orange, apple, blueberries, strawberries
**Fresh Bar C**
GC1: Spinach, mango, banana, orange
FC1: Strawberries, mint, lime, apple
FC2: Banana, strawberries, apple
FC3: Blueberries, raspberries, strawberries, pineapple, apple
**Fresh Bar D**
GD1: Spinach, orange, carrot, banana
FD1: Kiwi, orange, banana
FD2: Strawberries, banana, apple
**Fresh Bar E**
FE1: Apple, cucumber, spinach
FE2: Pear, beetroot, carrot, celery
FE3: Grapefruit, orange, ginger
**Fresh Bar F**
GF1: Spinach, pear, banana
FF1: Strawberries, banana, orange

**Table 2:** Concentrations of applied antibiotics according to EUCAST and CLSI [36,37].

	CFB	ENC
	EUCAST (mg/L)	
Ampicillin	9	9
Gentamicin	5	129
Chloramphenicol	9	-
Ciprofloxacin	1	5
Tetracycline	16 *	-
Vancomycin	-	5

CFB—coliform bacteria; ENC—enterococci. * CLSI resistance breakpoint.

**Table 3:** Number of total fungi and bacteria in the smoothie samples.

FSE	Type of Smoothie	Total Aerobic Bacteria	Yeasts and Fungi	Coliform Bacteria	Enterococci
		log CFU/mL			
A	GA1	3.3 ± 0.17	2.9 ± 0.12	2.7 ± 0.14	2.0 ± 0.06
	GA2	3.5 ± 0.11	2.7 ± 0.11	2.6 ± 0.10	2.9 ± 0.15
	FA1	3.3 ± 0.10	2.6 ± 0.13	2.8 ± 0.14	1.7 ± 0.07
	FA2	2.9 ± 0.09	2.9 ± 0.15	2.7 ± 0.08	2.1 ± 0.11
B	GB1	3.1 ± 0.16	2.8 ± 0.14	2.6 ± 0.09	1.7 ± 0.05
	GB2	3.1 ± 0.12	1.8 ± 0.05	2.9 ± 0.15	2.5 ± 0.13
	FB1	2.9 ± 0.09	2.8 ± 0.08	2.7 ± 0.16	2.2 ± 0.11
	FB2	3.0 ± 0.15	2.6 ± 0.09	2.9 ± 0.11	1.9 ± 0.06
C	GC1	3.2 ± 0.13	2.8 ± 0.11	2.1 ± 0.06	2.6 ± 0.13
	FC1	3.2 ± 0.16	2.4 ± 0.09	2.6 ± 0.13	1.1 ± 0.02
C	FC2	3.4 ± 0.17	2.7 ± 0.14	2.0 ± 0.08	ND
	FC3	3.2 ± 0.10	2.9 ± 0.09	2.1 ± 0.11	1.5 ± 0.05
D	GD1	7.2 ± 0.43	3.7 ± 0.15	3.7 ± 0.19	1.6 ± 0.08
	FD1	7.2 ± 0.50	3.3 ± 0.16	3.4 ± 0.17	ND
	FD2	7.3 ± 0.44	3.2 ± 0.10	3.3 ± 0.13	1.6 ± 0.09
E	FE1	5.9 ± 0.30	3.3 ± 0.17	3.8 ± 0.23	1.8 ± 0.05
	FE2	5.5 ± 0.28	2.9 ± 0.12	4.2 ± 0.25	2.0 ± 0.08
	FE3	5.8 ± 0.29	3.5 ± 0.18	2.2 ± 0.11	1.3 ± 0.04
F	GF1	5.5 ± 0.17	ND	2.2 ± 0.09	2.2 ± 0.11
	FF1	7.3 ± 0.37	3.3 ± 0.11	3.7 ± 0.19	2.5 ± 0.13

FSE—food service establishment; ND—not detected.

**Table 4:** Occurrence of antibiotic-resistant coliform bacteria in samples of smoothie.

FSE	Type of Smoothie	AMP	GEN	CIP	CHL	TET
		log CFU/mL				
A	GA1	2.2 ± 0.11	ND	ND	1.7 ± 0.09	1.5 ± 0.05
	GA2	1.3 ± 0.04	ND	ND	ND	ND
	FA1	ND	ND	ND	ND	ND
	FA2	ND	ND	ND	ND	ND
B	GB1	ND	ND	ND	ND	ND
	GB2	ND	ND	ND	ND	2.7 ± 0.14
	FB1	ND	ND	1.5 ± 0.06	ND	ND
	FB2	2.3 ± 0.07	ND	ND	1.7 ± 0.05	ND
C	GC1	1.7 ± 0.07	ND	ND	ND	ND
	FC1	2.5 ± 0.13	ND	ND	ND	ND
	FC2	1.9 ± 0.10	ND	ND	ND	ND
	FC3	1 ± 0.03	1.6 ± 0.05	ND	ND	ND
D	GD1	3.98 ± 0.12	2.87 ± 0.17	ND	ND	ND
	FD1	3.36 ± 0.13	3.21 ± 0.16	ND	2.67 ± 0.11	ND
	FD2	3.47 ± 0.21	3.14 ± 0.13	ND	ND	ND
E	FE1	3.76 ± 0.19	3.66 ± 0.11	ND	ND	ND
	FE2	4.17 ± 0.13	4.01 ± 0.20	ND	2.62 ± 0.13	1 ± 0.04
	FE3	1.85 ± 0.09	1.48 ± 0.04	1.48 ± 0.07	ND	ND
F	GF1	4.15 ± 0.17	3.33 ± 0.17	3.72 ± 0.11	3.53 ± 0.18	ND
	FF1	3.76 ± 0.11	2.64 ± 0.08	2.72 ± 0.14	3.2 ± 0.13	ND

AMP—ampicillin, GEN—gentamicin, CIP—ciprofloxacin, CHL—chloramphenicol, TET—tetracycline; FSE—food service establishment; ND—not detected.

**Table 5:** Occurrence antibiotic-resistant enterococci in the smoothie samples.

FSE	Type of Smoothie	AMP	GEN	CIP	VAN
		log CFU/mL			
A	GA1	1.9 ± 0.06	1.7 ± 0.07	ND	1.7 ± 0.06
	GA2	ND	ND	ND	ND
	FA1	ND	1.6 ± 0.08	ND	1.5 ± 0.05
	FA2	ND	ND	ND	ND
B	GB1	1.6 ± 0.05	ND	ND	ND
	GB2	2 ± 0.08	ND	ND	2.4 ± 0.12
	FB1	ND	ND	ND	ND
	FB2	ND	ND	ND	ND
C	GC1	ND	ND	ND	ND
	FC1	ND	ND	ND	ND
	FC2	ND	ND	ND	ND
	FC3	ND	ND	ND	ND
D	GD1	ND	ND	ND	ND
	FD1	ND	ND	ND	ND
	FD2	1.6 ± 0.05	ND	ND	ND
E	FE1	ND	ND	ND	ND
	FE2	1 ± 0.02	1 ± 0.05	ND	ND
	FE3	ND	ND	ND	1.3 ± 0.09
F	GF1	2.15 ± 0.11	2.66 ± 0.13	ND	ND
	FF1	2.28 ± 0.14	ND	2.15 ± 0.08	ND

AMP—ampicillin, GEN—gentamicin, CIP—ciprofloxacin, VAN—vancomycin; FSE—food service establishment; ND—not detected.

**Table 6:** Number of selected microorganisms in smoothie samples according to the pretreatment of raw materials.

	Control	Water	WS
Type of Microorganism	log CFU/mL		
TAB	6.09 ± 0.30 ^c^	5.49 ± 0.27 ^b^	4.49 ± 0.22 ^a^
TCB	3.22 ± 0.10 ^c^	2.39 ± 0.07 ^b^	1.49 ± 0.03 ^a^
AMP	3.41 ± 0.14 ^c^	2.50 ± 0.08 ^b^	1.75 ± 0.09 ^a^
CIP	1.36 ± 0.04 ^b^	0.16 ± 0.01 ^a^	0.23 ± 0.01 ^a^
YF	5.92 ± 0.30 ^c^	5.62 ± 0.28 ^b^	5.22 ± 0.21 ^a^

Control—unwashed ingredients; Water—washed under drinking tap water; WS—treatment with aqueous solution of Sanosil, TAB—total aerobic bacteria; TCB—total coliform bacteria; AMP—ampicillin-resistant coliform bacteria; CIP—ciprofloxacin-resistant coliform bacteria; YF—yeasts and fungi. ^a–c^—within a row with different superscript letters are significantly different (*p* < 0.05); (*n* = 4).

## Data Availability

Data of the current study are available from the corresponding author on reasonable request.
